# *MoSfl1* Is Important for Virulence and Heat Tolerance in *Magnaporthe oryzae*

**DOI:** 10.1371/journal.pone.0019951

**Published:** 2011-05-19

**Authors:** Guotian Li, Xiaoying Zhou, Lingan Kong, Yuling Wang, Haifeng Zhang, Heng Zhu, Thomas K. Mitchell, Ralph A. Dean, Jin-Rong Xu

**Affiliations:** 1 Department of Botany and Plant Pathology, Purdue University, West Lafayette, Indiana, United States of America; 2 College of Plant Protection, Northwest A&F University, Yangling, Shanxi, China; 3 Department of Pharmacology and Molecular Sciences, Johns Hopkins University School of Medicine, Baltimore, Maryland, United States of America; 4 Department of Plant Pathology, Ohio State University, Columbia, Ohio, United States of America; 5 Department of Plant Pathology, North Carolina State University, Raleigh, North Carolina, United States of America; University of Missouri-Kansas City, United States of America

## Abstract

The formation of appressoria, specialized plant penetration structures of *Magnaporthe oryzae*, is regulated by the *MST11*-*MST7*-*PMK1* MAP kinase cascade. One of its downstream transcription factor, *MST12*, is important for penetration and invasive growth but dispensable for appressorium formation. To identify additional downstream targets that are regulated by Pmk1, in this study we performed phosphorylation assays with a protein microarray composed of 573 *M. oryzae* transcription factor (TF) genes. Three of the TF genes phosphorylated by Pmk1 *in vitro* were further analyzed by coimmunoprecipitation assays. One of them, *MoSFL1*, was found to interact with Pmk1 *in vivo*. Like other Sfl1 orthologs, the MoSfl1 protein has the HSF-like domain. When expressed in yeast, *MoSFL1* functionally complemented the flocculation defects of the *sfl1* mutant. In *M. oryzae*, deletion of *MoSFl1* resulted in a significant reduction in virulence on rice and barley seedlings. Consistent with this observation, the *Mosfl1* mutant was defective in invasive growth in penetration assays with rice leaf sheaths. In comparison with that of vegetative hyphae, the expression level of *MoSFL1* was increased in appressoria and infected rice leaves. The *Mosfl1* mutant also had increased sensitivity to elevated temperatures. In CM cultures of the *Mosfl1* and *pmk1* mutants grown at 30°C, the production of aerial hyphae and melanization were reduced but their growth rate was not altered. When assayed by qRT-PCR, the transcription levels of the *MoHSP30* and *MoHSP98* genes were reduced 10- and 3-fold, respectively, in the *Mosfl1* mutant. *SFL1* orthologs are conserved in filamentous ascomycetes but none of them have been functionally characterized in non-*Saccharomycetales fungi*. MoSfl1 has one putative MAPK docking site and three putative MAPK phosphorylation sites. Therefore, it may be functionally related to Pmk1 in the regulation of invasive growth and stress responses in *M. oryzae*.

## Introduction

Rice blast caused by *Magnaporthe oryzae* is one of the most destructive diseases that affects rice production worldwide. In the last two decades, the *M. oryzae*-rice pathosystem has been developed as a model system for studying fungal-plant interactions [1], [2], [3]. The rice blast fungus can attack different tissues of rice plants although leaf blast and panicle blast cause the greatest yield losses. Plant infection is initiated when an asexual spore lands on the rice leaf surface and germinates. A specialized infection structure called an appressorium is formed at the tip of the germ tube. The fungus then uses enormous turgor pressure generated in the appressorium to penetrate the plant surface and underlying cells [4], [5]. Once inside plant cells, invasive hyphae grow biotrophically and spread to nearby cells, possibly *via* plasmodesmata [6], [7], before switching to necrotrophic growth later in the infection process.

Several signal transduction pathways have been implicated in the regulation of appressorium formation, penetration, and infectious growth in *M. oryzae* [3], [8]. The *PMK1* mitogen-activated protein (MAP) kinase, an ortholog of *Saccharomyces cerevisiae* Fus3/Kss1, is essential for appressorium formation and infectious growth in rice tissues [9]. The *pmk1* mutant fails to form appressoria and is nonpathogenic on healthy or wounded rice seedlings. However, germ tubes of the *pmk1* mutant have no defect in surface recognition and produce subapical swollen bodies on hydrophobic surfaces. The expression of *PMK1* is increased during appressorium formation and conidium development [10]. Pmk1 localizes to the nucleus in appressoria. In addition to the *PMK1* pathway, cyclic AMP signaling is known to be involved in the surface recognition and generation of appressorium turgor in *M. oryzae*. Exogenous cAMP stimulates appressorium formation on hydrophilic surfaces and deletion of the *MAC1* adenylate cyclase gene results in defects in appressorium formation [11], [12]. The *M. oryzae* genome has two genes encoding catalytic subunits of protein kinase A (PKA). The *cpkA* but not *cpk2* mutant is delayed in appressorium formation and defective in appressorium penetration [8], [13], [14]. Studies in several other phytopathogenic fungi also have shown that both the cAMP signaling and MAP kinase pathways are involved in the regulation of various plant infection and differentiation processes [15], [16]. However, molecular mechanisms regulating the interaction or cross-talking between these two pathways are not clear. In the corn smut fungus *Ustilago maydis*, the Prf1 transcription factor is activated by both the cAMP-PKA and MAP kinase pathways [17], [18].

In *M. oryzae*, several components of the *PMK1* pathway have been characterized, including *MST11*, *MST50*, and *MST12* [19], [20], [21]. *MST12*, an ortholog of yeast *STE12*, encodes a transcription factor that interacts with Pmk1 in yeast two-hybrid assays. Although it is defective in appressorial penetration and invasive growth, the *mst12* mutant still forms appressoria [22], [23]. To identify additional downstream targets that are regulated by Pmk1, here we performed *in vitro* phosphorylation assays with a protein microarray composed of recombinant proteins of 573 *M. oryzae* transcription factor (TF) genes that were individually expressed in the budding yeast [24]. Protein microarray has been shown as a powerful proteomics tool for profiling protein-protein, protein-DNA, protein-RNA, and protein-glycan interactions and for identification of substrates of various enzymes, such as protein kinases, acetyltransferases, ubiquitin E3 ligases [25], [26], [27]. Three of the putative Pmk1 targets identified in phosphorylation assays the *M. oryzae* TF microarray were further analyzed by coimmunoprecipitation assays. One of them, an ortholog of yeast *SFL1*, was found to interact with Pmk1 *in vivo*. In yeast, Sfl1 serves as a transcriptional repressor for the flocculation-related genes and a transcriptional activator for the stress-responsive genes [28], [29]. The *Mosfl1* deletion mutant was reduced in virulence and invasive growth but still formed appressoria. Similar to the *pmk1* mutant, it had increased sensitivity to elevated temperatures, suggesting that Sfl1 and Pmk1 may be functionally related in the regulation of invasive growth and stress responses in *M. oryzae*.

## Results

### MoSfl1 interacts with Pmk1

To identify transcription factors as the downstream effectors of Pmk1, a protein microarray composed of GST-fusion proteins of all predicted *M. oryzae* TF genes [24] was used for *in vitro* phosphorylation assays with purified recombinant proteins of Pmk1 [9], [30]. GST-Pmk1 fusion proteins are known to have protein kinase activities *in vitro* [9]. After incubating the protein microarray with GST-Pmk1 in the presence of r-^33^P-ATP for 30 min, the phosphorylation reaction was stopped and phosphorylation signals were analyzed with the GenePix Pro software [31], [32]. An autophosphorylation reaction without addition of any kinase was set up as a negative control. A total of 85 putative transcription factors were found to be phosphorylated by Pmk1.

Three of them, MGG_06971, MGG_09869, and MGG_04933, were selected for verification for their interactions with Pmk1 *in vivo*. MGG_06971 was named *MoSFL1* because it is orthologous to yeast *SFL1* [28]. MGG_09869 is homologous to *SWI6* in yeast [33] and MGG_04933 is a putative transcription factor conserved in filamentous fungi. FLAG-tagged constructs were generated for these three genes by the yeast GAP repair approach [34] and transformed into the wild-type strain 70-15. Transformants expressing the MGG_06971-, MGG_09869-, or MGG_04933-3×FLAG fusion constructs (Table 1) were identified by PCR and confirmed by western blot analysis (Fig. S1).

**Table 1:** Wild-type and mutant strains of *Magnaporthe oryzae* used in this study.

Strain	Genotype description	Reference
70-15	Wild type (*MAT1-1*, *AVR-Pita*)	[71]
Guy11	Wild type (*MAT1-2*, *avr-Pita*)	[71]
Ku80	*Mgku80* deletion mutant of Guy11	[37]
nn78	*pmk1* deletion mutant of Guy11	[9]
I-27	*cpkA* deletion mutant of 70-15	[14]
GSF7	Transformant of 70-15 expressing *MoSFL1*-3×FLAG fusion	This study
GSF9	Transformant of 70-15 expressing *MoSFL1*-3×FLAG fusion	This study
GWF7	Transformant of 70-15 expressing MGG_09869-3×FLAG fusion	This study
GWF9	Transformant of 70-15 expressing MGG_09869-3×FLAG fusion	This study
WDF1	Transformant of 70-15 expressing MGG_04933-3×FLAG fusion	This study
GK102	*Mosfl1*deletion mutant of Ku80	This study
GK116	*Mosfl1*deletion mutant of Ku80	This study
GK135	*Mosfl1*deletion mutant of Ku80	This study
C49	*Mosfl1/MoSFL1*-GFP complemented transformant	This study
C51	*Mosfl1/MoSFL1*-GFP complemented transformant	This study
P1	*MoSFL1* ^ΔT231A^ in GK102	This study
P2	*MoSFL1* ^ΔS474A^ in GK102	This study
P3	*MoSFL1* ^ΔT508A^ in GK102	This study

**Figure 1:**
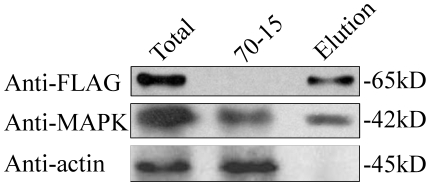
MoSfl1 co-immunoprecipitates with Pmk1. Western blots of total proteins (Total) and proteins eluted from the anti-FLAG M2 beads (Elution) of transformant GSF7 that expressed the *MoSFL1*-3×FLAG construct were detected with the anti-FLAG and anti-MAPK antibodies. Total proteins isolated from the wild-type strain (70-15) and detection with the anti-actin antibody was included as the control.

We then isolated total proteins from transformant GSF7 expressing the *MoSFL1*-3×FLAG construct and used the anti-FLAG antibody to pull down proteins interacting with MoSfl1 in *M. oryzae*. In both total proteins and proteins eluted from anti-FLAG M2 beads of transformant GSF7, a 64-kD band of the expected size of MoSfl1-3×FLAG fusion was detected with the anti-FLAG antibody (Fig. 1). When detected with an anti-MAPK antibody [10], the 42-kD Pmk1 band also was observed (Fig. 1). In the control experiment, the anti-actin antibody detected a 45-kD actin band only in total proteins isolated from 70-15 and transformant GSF7 but not in proteins eluted from anti-FLAG M2 beads. These data indicate that Pmk1 was co-immunoprecipitated with MoSfl1-3×FLAG.

Similar co-immunoprecipitation (co-IP) assays were conducted with proteins isolated from transformants GWF9 and WDF1 that contained the MGG_09869- and MGG_04933-3×FLAG fusion constructs, respectively. However, co-immunoprecipitation of Pmk1 with FLAG-tagged MGG_09869 and MGG_04933 proteins was not observed (data not shown), indicating that MoSfl1 but not MGG_09869 or MGG_04933 physically interacts with Pmk1 in *M. oryzae*.

### *MoSFL1* functionally complements the yeast *sfl1* mutant

*MoSFL1* shared 29% and 32% similarity with *S. cerevisiae SFL1* and *CaSFL1* of *Candida albicans*, respectively. It is highly similar to its putative orthologs in other filamentous ascomycetes, including *Fusarium graminearum* (60% identity) and *Neurospora crassa* (53% identity). Like Sfl1 and CaSfl1, MoSfl1 contains a heat shock factor [HSF] domain that binds to the inverted repeats of AGAA-n-TTCT known as heat shock element [35].

**Figure 2:**
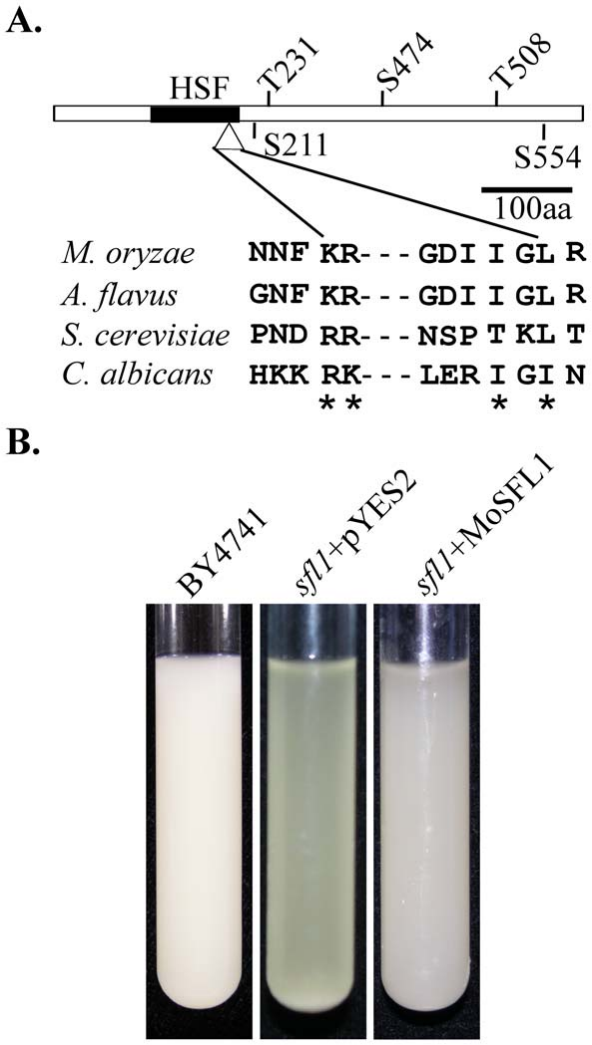
Structural elements of MoSfl1 and complementation assays with the yeast *sfl1* mutant. **A.** The MoSfl1 protein contains a heat shock factor (HSF) domain (black box), a MAPK docking site (triangle), three putative MAP kinase phosphorylation sites (T231, S474, and T508), and two putative PKA phosphorylation sites (S211 and S554). The amino acid sequence of the MAPK docking site is conserved among Sfl1 and its orthologs from *Magnaporthe oryzae*, *Aspergillus flavus*, and *Candida albicans*. **B.** Expression of *MoSFL1* suppressed the flocculation defect of the yeast *sfl1* mutant. Cultures of *Saccharomyces cerevisiae* wild type strain BY4741 (*SFL1*) and transformants of *Δsfl1* carrying pYES2 or pYES2-*MoSFL1* were shaken at 30°C for 16 h and kept still for 15 min before being photographed.

In comparison with CaSfl1, MoSfl1 does not possess glutamine-rich regions that are likely involved in protein-protein interactions. However, MoSfl1 has one MAP kinase docking site (amino acid residues KRGDIIGL) and three putative MAP kinase phosphorylation sites (Fig. 2A) that are conserved in the MoSfl1 orthologs from other fungi. To determine its function in *S. cerevisiae*, the *MoSFL1* ORF was cloned into pYES2 as pYES2-*MoSFL1* and transformed into the *sfl1* mutant in the BY4741 yeast strain background [36]. Five Ura^+^ transformants were confirmed by PCR to contain the pYES2-*MoSFL1* construct. Although only data with one of these transformants are provided, all of them were complemented in the flocculation defects. After overnight incubation in the galactose-containing medium, the *sfl1* mutant cultures were clear due to the sediment of yeast cells. Like the wild type, cultures of the *sfl1*/*MoSFL1* transformants were turbid (Fig. 2B). These results indicate that *MoSFL1* can functionally complement the *sfl1* mutant when expressed in *S. cerevisiae*.

### The *Mosfl1* mutant is reduced in conidiation but has a normal growth rate

**Figure 3:**
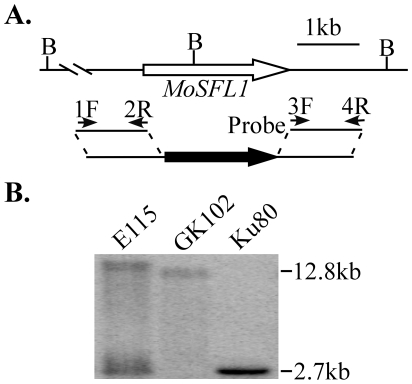
The *MoSFL1* gene replacement vector and mutants. **A.** The *MoSFL1* genomic region and gene replacement construct. The upstream and downstream flanking sequences were amplified with primers 1F/2R and 3F/4R, respectively, and connected with the *hph* cassette by double joint PCR. B, *Bam*HI. **B.** Southern blot of *Bam*HI-digested DNA of Ku80, *Mosfl1*mutant GK102, and ectopic transformant E115 was hybridized with the downstream sequence of *MoSFL1* amplified with primers 3F and 4R as the probe. Because of the *Bam*HI site located in the middle of the *MoSFL1* gene, the *Mosfl1* deletion mutant had a 12.8-kb band instead of the wild-type 2.7-kb band.

To knock out *MoSFL1*, its upstream and downstream flanking sequences were amplified with primer pairs 1F/2R and 3/4R (Fig. 3A). The *MoSFL1* gene replacement construct (Fig. 3A) was then generated with the double-joint PCR method and transformed into *M. oryzae* strain Ku80 [37]. Around 140 hygromycin-resistant transformants were isolated and screened by PCR with primers ScF and ScR. Three putative *Mosfl1* gene replacement mutants (Table 1) were identified and confirmed by Southern blot analysis (Fig. 3), indicating that the gene replacement frequency was approximately 2.1% for *MoSFL1* even in the Ku80 [37] strain background. When genomic DNAs were digested with *Bam*HI and hybridized with the downstream flanking sequence of the *MoSFL1* gene as the probe, the *Mosfl1* knockout mutant had a 12.8-kb band instead of the 2.7-kb wild-type band (Fig. 3B). The ectopic transformant E115 had the 2.7-kb wild-type band and an additional band (>12.8 kb) resulting from ectopic integration of the gene replacement cassette in the genome.

In comparison with Ku80, the *Mosfl1* mutant was reduced about 70% in conidiation (Table 2). In contrast, the growth rate of the Mosfl1 mutant was similar to that of Ku80 or ectopic transformant E115 (Table 2). To determine whether deletion of *MoSFL1* had any effect on responses to different physiological stresses, we assayed vegetative growth of the mutant on various media. The *Mosfl1* mutant had a growth rate similar to that of Ku80 under nitrogen- or carbon-starvation conditions, on V8 juice agar and minimal medium (MM), and on complete medium (CM) with 1 M sorbitol, 0.7 M NaCl, or different concentrations of H_2_O_2_ (Table S1). These results indicate that *MoSFL1* is dispensable for responses to nutritional, hyperosmotic, and oxidative stresses.

**Table 2:** Phenotypes of the *Mosfl1* mutant in growth, development, and differentiation.

Strain	Conidiation(10^6^ spores/plate)	Growth rate(mm/day)^a^	Conidial germination (%)^b^	Appressoriumformation (%)	Cytorrhysis^c^
Ku80	101.7±16.4	3.3±0.1	100±0.0	95.2±2.4	63±3.6
E115	100.0±16.9	3.4±0.1	N/A^d^	N/A	N/A
GK102	28.7±8.9	3.4±0.1	100±0.0	96.9±1.2	73.8±0.5
C49	105.3±1.2	3.5±0.1	N/A	N/A	N/A

a The growth rate was measured with 14-day-old CM cultures grown in race tubes. Mean and standard deviation were calculated with results from three replicates.

b Percentage of conidia germination and appressorium formation by 24 h. Means and standard errors were calculated from three independent repeats (at least 100 conidia were counted in each repeat).

c Percentage of appressoria that underwent cytorrhysis in 35% PEG. Mean and standard deviations were calculated from four replicates. At least 100 appressoria were examined in each repetition.

d Not assayed.

### *MoSFL1* is dispensable for conidium germination, appressorium formation, and turgor generation

To determine the role *MoSFL1* in infection-related morphogenesis, we assayed appressorium formation with the *Mosfl1* mutant. On hydrophobic surfaces, the *Mosfl1* mutant had no defect in surface attachment or conidium germination. After 2 h of incubation at room temperature, over 95% of mutant conidia germinated and produced normal germ tubes (data not shown). A vast majority of the *Mosfl1* germ tubes (>95%) formed appressoria within 24 h. We also examined appressorium formation at 8 h. The wild-type and mutant strains had no obvious difference in the percentage of germ tubes forming appressoria. These data indicate that *MoSFL1* is dispensable for conidium germination and appressorium formation.

In *M. oryzae*, appressorium turgor plays a critical role in plant penetration. To determine whether the *Mosfl1* mutant had defects in turgor generation, we conducted the cytorrhysis assay [4]. In the presence of 35% PEG, Ku80 and the *Mosfl1* mutant strains had similar amounts of appressoria that underwent cytorrhysis (Table 2, *P*>0.1), suggesting that appressoria of the wild-type and mutant strains had similar turgor pressures.

### The *Mosfl1* mutant is reduced in virulence

**Figure 4:**
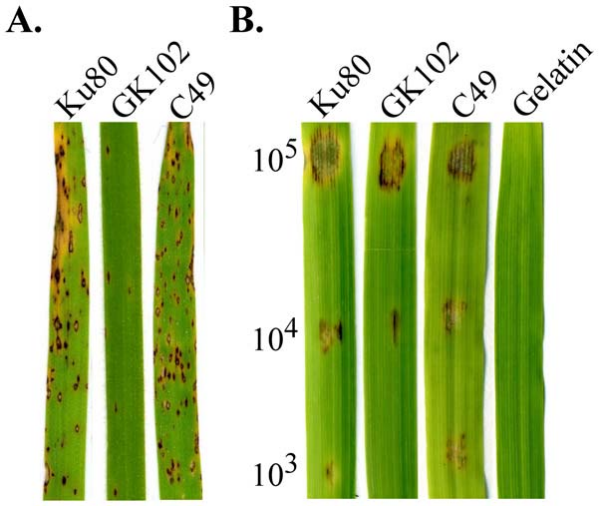
Infection assays with the *Mosfl1* mutant. **A.** Seedlings of rice cultivar CO-39 were sprayed with conidia of Ku80, *Mosfl1* mutant GK102, and complemented transformant C49. Typical leaves were photographed 7 days post inoculation (dpi). **B.** Detached barley leaves were drop inoculated with conidia from the same set of strains. The concentrations of conidium suspensions (conidia/ml) were marked on the left. Inoculation with 0.25% gelatin was the negative control.

To determine the virulence of the *Mosfl1* mutant, two-week-old seedlings of rice cultivar CO-39 were used for infection assays. On rice leaves sprayed with conidium suspensions of 5×10^4^ spores/ml, strain Ku80 formed typical blast lesions (Fig. 4A). The *Mosfl1* deletion mutant caused fewer and often smaller lesions on rice leaves. On average, 3.3±0.6 lesions/5-cm leaf tip were observed on leaves sprayed with the *Mosfl1* mutant. Under the same conditions, the wild type caused 21.3±3.5 lesions/5-cm leaf tip. Even after prolonged incubation such as 10 dpi, lesions caused by the mutant were still restricted and normally lacked the necrosis zone on the edge (Fig. 4A). To confirm that the observed phenotypes are directly related to *MoSFL1* deletion, we generated the *MoSFL1*-GFP fusion construct and transformed it into the *Mosfl1* mutant GK102. The resulting transformant C49 (Table 1) was normal in conidiation (Table 2) and plant infection (Fig. 4).

To further prove that the *Mosfl1* mutant is reduced in virulence, detached barley leaves were drop inoculated with different concentrations of conidia. At 1×10^3^ conidia/ml, only the sites inoculated with the wild type but not the *Mosfl1* mutant had visible symptoms (Fig. 4B). At 1×10^4^ conidia/ml, the *Mosfl1* mutant caused limited necrosis at the inoculation sites. The wild type caused much more severe symptoms under the same conditions (Fig. 4B). Even at the sites inoculated with 1×10^5^ conidia/ml, the wild type still appeared to be more virulent than the *Mosfl1* mutant. Extensive necrosis surrounding the drop-inoculation sites was only observed in the wild type (Fig. 4B). These results indicate that the *Mosfl1* mutant had a reduced virulence on rice and barley leaves and was defective in lesion expansion or development of the surrounding necrosis zone.

### *MoSFL1* plays a role in infectious growth after penetration

**Figure 5:**
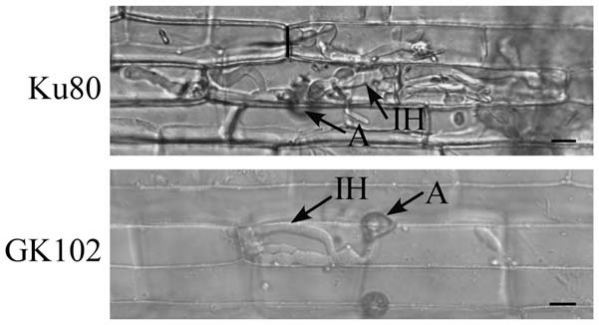
Penetration assays with the *Mosfl1* mutant. Rice leaf sheaths inoculated with conidia from Ku80 and *Mosfl1* mutant GK102 were examined 48 hpi. The mutant was restricted in invasive growth compared with the wild type. A, appressorium; IH, invasive hyphae. Bar = 10 µm.

Because the *Mosfl1* mutant formed smaller lesions than the wild type on rice and barley leaves, we examined the defects of the *Mosfl1* mutant in infectious growth after penetration. In penetration assays with rice leaf sheath epidermal cells, invasive hyphae formed by Ku80 branched and spread to nearby rice leaf sheath epidermal cells by 48 h (Fig. 5). Under the same conditions, the mutant normally had only limited growth. At 48 h, invasive hyphae of the *Mosfl1* mutant appeared to be confined to the penetrated plant cell. Extensive growth of invasive hyphae was only observed in plant tissues inoculated with Ku80 but not the *Mosfl1* mutant.

### The *Mosfl1* mutant is defective in heat tolerance

**Figure 6:**
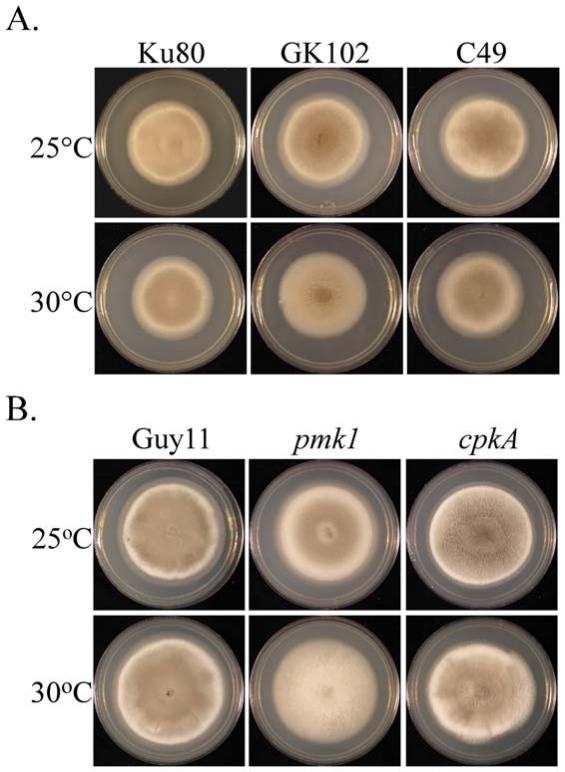
Increased heat sensitivity in the *Mosfl1* and *pmk1* mutants. **A.** Five-day-old CM cultures of Ku80, *Mosfl1* mutant GK102, and complemented transformant C49. **B.** Seven-day-old CM cultures of the wild type Guy11, *pmk1* mutant nn78, and *cpkA* mutant I-27. All of the cultures were incubated at 25°C (top) or 30°C (bottom). While the growth rates of Ku80 and Guy11 were not affected, the production of aerial hyphae was reduced in the *Mosfl1* and *pmk1* mutants when cultured at 30°C.

Similar to Sfl1 of *S. cerevisiae*, MoSf1 has a heat-shock factor domain and may be involved in regulating heat tolerance. When cultured at 25°C, the *Mosfl1* mutant and the Ku80 strain had similar colony morphologies (Fig. 6A). However, colonies of the *Mosfl1* mutant were significantly reduced in aerial hyphal growth compared with those of Ku80 when cultured at 30°C on CM (Fig. 6A) or oatmeal agar (Fig. S2) plates. Aerial hyphal growth of the complemented transformant C49 was similar to that of Ku80 at 30°C, suggesting that MoSfl1 plays a role in heat tolerance during the growth of aerial hyphae. We also assayed heat tolerance with the *pmk1* and *cpkA* mutants. Similar to the *Mosfl1* mutant, the *pmk1* mutant was significantly reduced in the production of aerial hyphae at 30°C (Fig. 6B). The elevated temperature had no obvious effect on the *cpkA* mutant (Fig. 6B).

To confirm that the observed phenotypes are directly related to *MoSFL1* deletion, we generated the *MoSFL1*-GFP fusion construct and transformed it into mutant GK102. The resulting transformant C49 (Table 1) was normal in conidiation (Table 2), plant infection (Fig. 4), and growth at 30°C (Fig. 6A), indicating that expression of the wild-type *MoSFL1* allele fully complemented the defects of the *Mosfl1* mutant.

### Downstream genes regulated by *MoSFL1*

**Figure 7:**
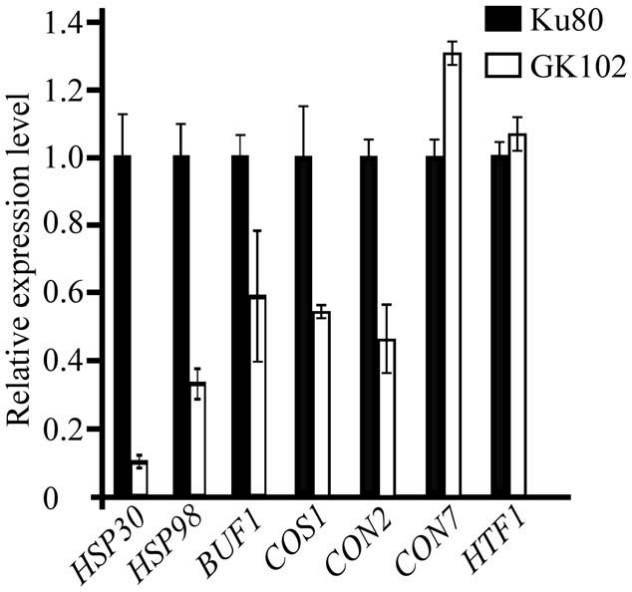
qRT-PCR assay of *MoHSP30*, *MoHSP98*, *BUF1*, *COS1*, *CON2*,*CON7*, and *HTF1* expression in the Ku80 and *Mosfl1* mutant strains. The relative expression levels of *MoHSP30*, *MoHSP98*, *BUF1*, *COS1*, *CON2*, *CON7*, and *HTF1* were compared between the GK102 and Ku80 strains (arbitrarily set to 1) cultured at 30°C. Mean and standard error were calculated with data from three biological replicates.

Because of the defect of the *Mosfl1* mutant in heat tolerance and the regulation of heat shock-related genes by Sfl1 in yeast and *C. albicans* [29], we assayed the transcript abundance of *M. oryzae* genes homologous to yeast *HSP30*, *HSP60*, and *HSP98* that encode heat shock proteins. RNA samples were isolated from cultures of Ku80 and mutant GK102. Compared to Ku80, the transcription levels of *MoHSP30* and *MoHSP98* were reduced 10- and 3-fold, respectively, in the *Mosfl1* mutant (Fig. 7). The expression of *MoHSP60* was not significantly changed in the mutant (data not shown).

On CM plates, colonies of the *Mosfl1* mutant appeared to be whitish or less pigmented compared to the grayish colonies of the wild type, which may be related to a reduction in melanin synthesis. In *M. oryzae*, one of the major genes involved in the biosynthesis of melanin is *BUF1* [38]. When assayed by qRT-PCR, the transcription level of *BUF1* was reduced about 2-fold in the *Mosfl1* mutant (Fig. 7). These results suggest that MoSfl1 is involved in the transcriptional regulation of melanin biosynthesis and some heat-shock related genes, such *HSP30* and *HSP98* homologs in *M. oryzae*.

Because the *Mosfl1* mutant reduced in conidiation, we also assayed the expression levels of four genes that are known to be related to conidiation, *COS1*, *CON2*, *CON7*, and *HTF1* [39], [40], [41]. While the expression of *CON7* and *HTF1* were not significantly altered in the *Mosfl1* mutant, the transcription levels of *COS1* and *CON2* were reduced over 2-fold in the *Mosfl1* mutant compared to Ku80 (Fig. 7). Because *COS1* is essential for conidiophore development [39], a reduction in *COS1* expression may be related to reduced conidiation in the mutant.

### *MoSFL1* expression is increased during plant infection compared with vegetative growth

Although expressing the *MoSFL*-GFP construct rescued the defects of the *Mosfl1* mutant in plant infection, conidiation, and heat tolerance, we failed to detect GFP signals in the conidia, appressoria, vegetative hyphae, or invasive hyphae of transformant C49 (data not shown). MoSfl1 has a nuclear localization signal and is predicted to be a nuclear protein. In *C. albicans*, CaSfl1 was shown to be localized to the nucleus [42], [43]. However, we failed to detect GFP signals in the nucleus in transformant C49.

**Figure 8:**
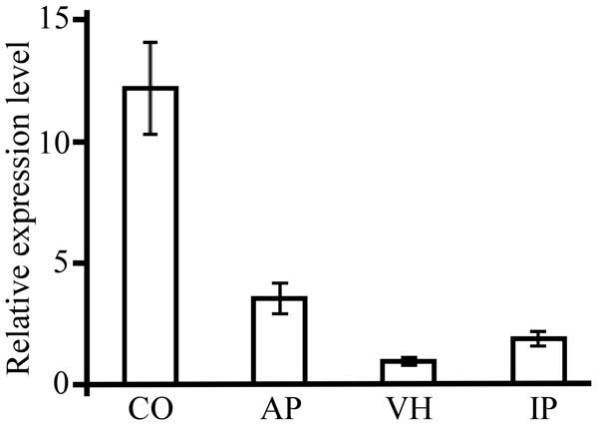
Expression of *MoSFL1* in different developmental stages. RNA samples used for qRT-PCR assays were isolated from conidia (CO), appressoria (AP), vegetative hyphae (VH), and infected plants (IP). The relative expression level of *MoSFL1* was compared to that of the VH stage (arbitrarily set to 1). Mean and standard error were calculated with data from three biological replicates.

We then used the qRT-PCR assay to estimate the expression levels of *MoSFL1* in conidia, mature appressoria, vegetative hyphae, and infected rice leaves (5 dpi). *MoSFL1* was expressed in all four stages (Fig. 8). The expression of *MoSFL1* was significantly increased in appressoria and infected rice leaves compared to vegetative hyphae. However, the highest transcription level of *MoSFL1* was in conidia. Nevertheless, GFP signals were not observed in conidia or appressoria of the *MoSFL1*-GFP transformant, which suggests that the activation of MoSfl1 and its localization to the nucleus are too transient to be detected with the GFP marker at these developmental stages. However, it remains possible that the expression level of *MoSFL1*, even in conidia, is relatively low compared to other genes.

## Discussion

The protein microarray of *M. oryzae* contains GST-fusion proteins of predicted TF genes in the genome [24]. In this study, we used the *M. oryzae* protein microarray for *in vitro* phosphorylation assays with GST-Pmk1 fusion proteins with kinase activities [9]. The *PMK1* MAP kinase and its orthologs are important for appressorium formation and invasive growth in *M. oryzae* [9], [19], [20]. A total of 85 genes was found to be phosphorylated by Pmk1, including homologs of Pro1 [44], StuA [45], *SFL1* [28], *PPR1* [46], and *SWI6* [47]. A number of fungal-specific Zn (2)–Cys (6) binuclear cluster and zinc finger proteins [48] also were found to be phosphorylated by Pmk1. However, the specificity of protein kinase-substrate interactions is likely compromised in *in vitro* phosphorylation assays. Many of these putative targets of Pmk1 identified in protein microarray experiments may be not functionally related with Pmk1 *in vivo*.

Three genes, MGG_04933, MGG_06971, and MGG_09869, were selected for co-IP assays. Only one of them, *MoSFL1* (MGG_06971), was confirmed by co-IP assays to interact with Pmk1 *in vivo*. In *S. cerevisiae*, Sfl1 functions as a transcriptional repressor of flocculation-related genes [28], [49]. Although the overall sequence identity is only 29%, *MoSFL1* could functionally complement the flocculation defects of the *sfl1* mutant (Fig. 1). One of the yeast genes regulated by Sfl1 is *FLO11*, which encodes a cell surface glycoprotein required for flocculation and filamentation [49], [50]. Transcription factor Flo8 functions antagonistically with Sfl1 as a transcriptional activator of *FLO11* [51], [52]. In *C. albicans*, *CaFLO8* and *CaSFL1* had opposite roles in hyphal development. *CaSFL1* also appeared to function as a negative regulator of flocculation [42], [43]. However, like many other filamentous ascomycetes, the *M. oryzae* genome lacks distinct orthologs of *FLO8* and *FLO11*. Therefore, the subset of genes regulated by *MoSFL1* or its function in *M. oryzae* may differ significantly from that of Sfl1 in yeast.

Deletion of *MoSFL1* had no obvious effect on responses to hyperosmotic, oxidative, and nutritional stresses. However, the *Mosfl1* mutant had increased sensitivities to elevated temperatures for aerial hyphal growth. At 30°C, aerial hyphal growth was reduced in the mutant. Like other Sfl1 orthologs, MoSfl1 has a HSF domain that recognizes heat shock elements in the promoters of target genes [35]. In yeast, genes with heat shock elements, such as *STA1* and *SUC2*, are repressed by Sfl1 [49], [53], [54]. However, Sfl1 also is involved in the activation of *HSP30* transcription [29]. When assayed by qRT-PCR, the expression levels of *MoHSP30* and *MoHSP98* genes were significantly reduced in the *Mosfl1* mutant. In *C. albicans*, CaSfl1 also is involved in the activation of heat-shock protein genes *HSP30* and *HSP90* under certain stress conditions [55]. *SFL1* orthologs may be well conserved in the activation of heat shock related genes in ascomycetes.

To date, *SFL1* orthologs have not been functionally characterized in plant pathogenic fungi. Deletion of *MoSFL1* had no effect on hyphal growth *in vitro*, which is different from the *Agsfl1* mutant in *Ashbya gossypii* [56]. Compared to the parental Ku80 strain, deletion of *MoSFL1* had no obvious effect on appressorium formation or turgor pressure production. The *Mosfl1* mutant was reduced in conidiation (Table 2) and virulence (Fig. 4A). In comparison with Ku80, the *Mosfl1* mutant produced fewer lesions and the lesions caused by the mutant were usually smaller than those of the wild type. Reduced pathogenicity of the *Mosfl1* mutant is likely related to its defects in invasive hyphal growth as shown in penetration assays with rice leaf sheaths (Fig. 5). In *C. albicans*, CaSfl1 represses the expression of several hypha-specific genes, including *HWP1*, *ECE1*, *ALS1*, *ALS3*, and *FLO8* [43], [55]. Either overexpression or deletion of *CaSFL1* attenuated the virulence of *C. albicans* in the mouse model. In *M. oryzae*, *MoSFL1* may be involved in the repression of genes that are detrimental to *in planta* biotrophic growth, such as genes encoding cell wall degrading enzymes or enzymes responsible for the synthesis of phytotoxic compounds. It is also possible that *MoSFL1* negatively regulates genes only required for vegetative growth. Deletion of *MoSFL1* may result in improper regulation of subsets of genes and a reduction in conidiation and virulence.

Similar to *MST12*, *MoSFL1* is involved in invasive hyphal growth. Although the *Mosfl1* mutant was reduced in *BUF1* expression in vegetative hyphae, it still formed melanized appressoria. Melanin biosynthesis is regulated differently in appressoria and in vegetative hyphae in *M. oryzae* and *Colletotrichum lagenarium* [57]. Our results indicated that melanization was normal in appressoria but reduced in vegetative hyphae in the *Mosfl1* mutant. In *M. oryzae*, *PTH12* and *CON7* are two transcription factor genes that are reported to be essential for appressorium formation [58], [59], suggesting that they may be functionally related with the Pmk1 pathway. However, none of them were identified in the phosphorylation assays with the *M. oryzae* protein arrays with Pmk1. Also, we have generated the *pth12* and *con7* deletion mutants. Unlike the *pmk1* mutant [41], hyphal tips of the *pth12* and *con7* mutants, had no defects in appressorium formation (Kong and Xu, unpublished).

MoSfl1 has one putative MAPK docking site, three putative MAPK phosphorylation sites, and two PKA phosphorylation sites that are conserved in Sfl1 orthologs from other filamentous ascomycetes. In *S. cerevisiae*, *FLO11* was regulated by both MAP kinase and cAMP filamentation signaling pathways through transcription factors Ste12, Tec1, Flo8, and Sfl1 [49], [52]. There are no distinct orthologs of Flo8 and Tec1 in *M. oryzae*. Therefore, the MoSfl1-related regulatory network in *M. oryzae* must be different from that of *S. cerevisiae*. To test whether MoSfl1 functions downstream from Pmk1, we generated *MoSFL1* mutant alleles in which three putative MAPK phosphorylation sites were deleted, and transformed these constructs into mutant GK102. The resulting transformants were normal in virulence (data not shown), suggesting that deletion of individual MAPK phosphorylation sites had no effect on its complementation of the *Mosfl1* mutant. Therefore, these putative MAP phosphorylation sites are either not important or play redundant roles with other MAPK or PKA phosphorylation sites in the regulation of MoSfl1. In *S. cerevisiae* and *C. albicans*, Sfl1 proteins are phosphorylated by the Tpk2 catalytic subunit of PKA [51]. Although MoSfl1 was not phosphorylated by CpkA *in vitro* and the *Mosfl1* and *cpkA* mutants had distinct phenotypes, it remains possible that the two putative PKA phosphorylation sites of MoSfl1 play a role in its activation. In *C. albicans*, the nuclear localization of CaSfl1 was not altered in the *tpk2* null mutant or in response to exogenous cAMP, suggesting that other signaling pathways (possibly a MAP kinase pathway) also are involved in the activation of CaSfl1 [43]. In *U. maydis*, the Prf1 transcription factor is functionally related to the cAMP-PKA and Kpp2/Kpp6 MAP kinase pathways [17], [18]. It is possible that MoSfl1 is regulated by both the Pmk1 MAP kinase and cAMP signaling pathways in *M. oryzae*. Further characterization of the activation of MoSfl1 may lead to better understanding of the interaction between these two important signal transduction pathways.

## Materials and Methods

### Strains and growth conditions

The *M. oryzae* wild-type and mutant strains (Table 1) were routinely cultured on oatmeal agar (OTA) and complete medium (CM) as described previously [60]. For the heat tolerance assay, CM cultures were incubated at 30°C or 25°C for 7 days. Assays for growth rate and conidiation were conducted with OTA cultures [61]. Protoplast preparation and transformation were performed as described [62]. Transformants were selected on medium with 250 µg/ml of hygromycin B or 200 µg/ml zeocin (Invitrogen, CA).

### Phosphorylation assays with the *M. oryzae* protein microarray

The protein microarray composed of all predicted *M. oryzae* transcription factors [24] were used for *in vitro* phosphorylation assays following the procedure described in phosphorylation assays with the *S. cerevisiae* protein microarray [31], [32]. GST-Pmk1 fusion proteins were expressed in the budding yeast. Purified GST-Pmk1 was tested for protein kinase activity *in vitro* using myelin basic protein (MBP) as the substrate [9], [31]. The *M. oryza*e protein microarray was incubated with GST-Pmk1 in the presence of r-^33^P-ATP at 30°C for 30 min [31]. The phosphorylation reaction was stopped with 0.5% SDS. After washing and drying, the microarray was exposed to an X-ray film. Phosphorylation signals were analyzed with the GenePix Pro software (www.moleculardevices.com). Positives were identified with a cutoff value of 3 [31], [32].

### Complementation of the yeast *sfl1* mutant

The *MoSFL1* ORF was amplified with primers YCF/YCR (Table S2) from the first strand cDNA of Guy11 and cloned into plasmid pGEM-T Easy (Promega, WI). Among the resulting clones sequenced, clone pGT25 contains the entire ORF of *MoSFL1*, which was then sub-cloned into pYES2 as pMoSFL1. Competent cells of the *sfl1* mutant of *S. cerevisiae* (Open Biosystems, AL) were prepared and transformed with pMoSFL1 using the alkali-cation yeast transformation kit (MP Biomedicals, OH). Ura3^+^ transformants were confirmed by PCR and assayed for flocculation in YPGal liquid medium as described previously [43], [63]. The yeast strain BY4741 (*MATa his3 leu2 met15 ura3*) used to generate the *sfl1* mutant was used as the control.

### Molecular manipulations

Putative MoSfl1-regulating genes were assayed by qRT-PCR. RNAs from conidia, appressoria, vegetative hyphae, and infected rice tissues were extracted with TRIzol (Invitrogen, CA) and reverse transcription was done with the AccuScript High Fidelity 1st Strand cDNA Synthesis Kit (Agilent Technologies, CA). RT-PCR was performed with the Stratagene Gene MX 3000 PM using the 2×Brilliant SYBR Green QPCR master mix (Agilent Technologies, CA). The relative quantification of each transcript was calculated by the 2-^ΔΔ^CT method [64] using the tubulin gene (MGG_00604) as the internal control.

### Co-immunoprecipitation assays

The *MoSFL*-3×FLAG fusion was constructed by cloning the PCR products amplified with primers 6971flag/1F and 6971flag/3R from genomic DNA and co-transformed with *Xho*I-digested pHZ126 into XK1-25 [34] as pGL12. Plasmid pGL12 was transformed into the wild-type strain 70-15. Transformants expressing the *MoSFL1*-3×FLAG construct were identified by PCR screens and confirmed by western blot analysis using anti-FLAG antibodies. Total proteins were extracted from these confirmed transformants and incubated with anti-FLAG M2 affinity gel (Sigma-Aldrich, MO) at 4°C overnight. Proteins bound to anti-FLAG beads were eluted and detected with anti-FLAG (Sigma-Aldrich, MO), anti-actin (Sigma-Aldrich, MO), or anti-MAPK (Cell Signaling Technology, MA) as described previously.

### Generation of the *MoSFL1* gene replacement construct and mutants

The double-joint PCR method [65] was used to generate the *MoSFL1* gene replacement vector. A 0.8-kb upstream and a 0.8-kb downstream flanking sequences of *MoSFL1* were amplified with primer pairs 1F/2R and 3F/4R, respectively. The *hph* cassette was amplified with primers HYG/F and HYG/R from pCB1003. The products of double-joint PCR were transformed into protoplasts of Ku80 [37]. Putative *Mosfl1* deletion mutants were screened by PCR and confirmed by Southern blot analysis. For complementation assays, the wild-type allele of *MoSFL1* was amplified with primers SFG/1F and NG/R and cloned into pYF1 plasmid [66] with bleomycin resistance. The same yeast GAP repair approach [34] was used to generate the T231A, S474A, and T508A mutant alleles of *MoSFL1* that carried substitution mutations in three putative MAPK phosphorylation sites. The complementation vector and mutant alleles of *MoSFL1* were transformed into protoplasts of GK102.

### Germination, appressorium formation, cytorrhysis and penetration assays

Conidia were collected from one-week-old oatmeal agar cultures and resuspended in sterile distilled water to the concentration of 5×10^4^ conidia/ml. Conidial germination and appressorium formation were assayed with plastic cover slips (Fisher Scientific Co.) as described [67]. Appressorium turgor pressure was estimated by the incipient cytorrhysis method with solutions of PEG-8000 [4]. For penetration assays with rice leaf sheaths, conidia were suspended to 10^5^/ml and used to inoculate leaf sheaths of 3-week-old plants [6], [68]. Penetration and invasive hyphae were examined 48 h post inoculation by DIC microscopy.

### Plant infection assays

Two-week-old seedlings of the rice cultivar CO-39 and 10-day-old seedlings of the barley cultivar Golden Promise were used for infection assays. Conidia were collected from 10-day-old oatmeal agar cultures and resuspended to 5×10^4^/ml in 0.25% gelatin. Plant incubation, inoculation, and lesion examination were performed as described [13], [69]. The average number of lesions formed on the 5-cm tip regions of the second leaves of rice seedlings were counted as described [21], [70]. For drop-inoculation assays with detached barley leaves, conidia were diluted to 10^5^, 10^4^, and 10^3^/ml. A 15-µl drop of each dilution was pipetted onto barley leaves placed over 2% (w/v) water agar and kept in a sealed moisture chamber. Symptom development was examined after incubating at 25°C (12 h light/12 h dark) for 5 days.

## Supporting Information

Figure S1 **Western blot analysis with transformants GWF9 and WDF1.** Total proteins were isolated from a wild-type strain (70-15), transformant GWF9 expressing MGG_09869-3×FLAG, and transformant WDF1 expressing MGG_04933-3×FLAG fusion.
(TIF)

Figure S2 **Increased heat sensitivity of the** ***Mosfl1*** **mutant.** Five-day-old oatmeal agar cultures of Ku80, *Mosfl1* mutant GK102, and complemented transformant C49 grown at 30°C. The production of aerial hyphae was reduced in the *Mosfl1* mutant.
(TIF)

Table S1 Stress responses in the Mosfl1 mutants
(DOC)

Table S2 PCR primers used in this study
(DOC)
