# Supplementary figures and images for: MoSfl1 Is Important for Virulence and Heat Tolerance in Magnaporthe oryzae
Source: PLoS One. 2011 May 19;6(5):e19951. doi: 10.1371/journal.pone.0019951 (PMC3098271; doi:10.1371/journal.pone.0019951)

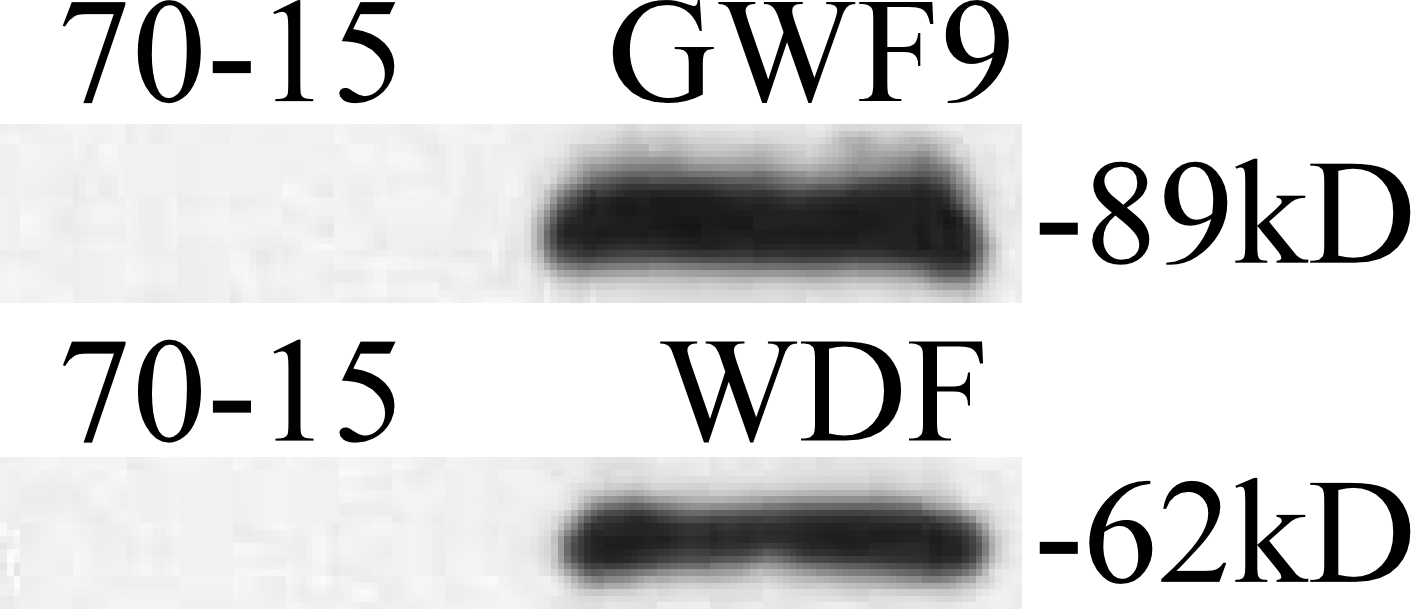

Supplement: Figure S1 — Western blot analysis with transformants GWF9 and WDF1. Total proteins were isolated from a wild-type strain (70-15), transformant GWF9 expressing MGG_09869-3×FLAG, and transformant WDF1 expressing MGG_04933-3×FLAG fusion. (TIF) [file pone.0019951.s001.tif]

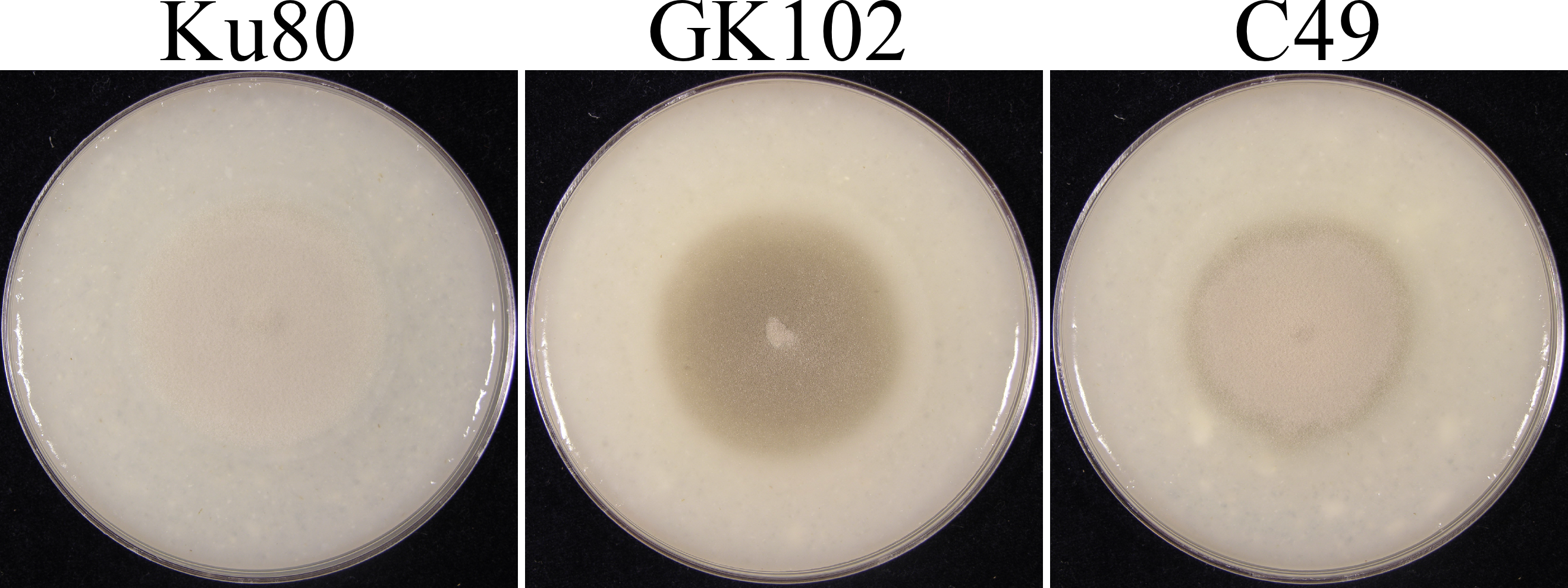

Supplement: Figure S2 — Increased heat sensitivity of the Mosfl1 mutant. Five-day-old oatmeal agar cultures of Ku80, Mosfl1 mutant GK102, and complemented transformant C49 grown at 30°C. The production of aerial hyphae was reduced in the Mosfl1 mutant. (TIF) [file pone.0019951.s002.tif]
