# Supplementary material for: MoSfl1 Is Important for Virulence and Heat Tolerance in Magnaporthe oryzae
Source: PLoS One. 2011 May 19;6(5):e19951. doi: 10.1371/journal.pone.0019951 (PMC3098271; doi:10.1371/journal.pone.0019951)
Supplement: Table S1 — Stress responses in the Mosfl1 mutants (DOC) [file pone.0019951.s003.doc]

**Table S1. Stress responses in the *Mosfl1* mutants^a^**

|  | Ku80  (mm/day) | E115  (mm/day) | GK102  (mm/day) |
| --- | --- | --- | --- |
| CM | 3.5±0.2 | 3.5±0.1 | 3.5±0.1 |
| CM+0.7M NaCl | 1.5±0.2 | 1.5±0.1 | 1.6±0.1 |
| CM+1M sorbitol | 2.0±0.1 | 2.1±0.1 | 2.2±0.1 |
| CM+1mM H_2_O_2_ | 2.9±0.1 | 2.8±0.1 | 2.8±0.1 |
| CM+3mM H_2_O_2_ | 2.5± 0.1 | 2.5±0.0 | 2.4±0.2 |
| CM+5mM H_2_O_2_ | 2.3± 0.1 | 2.3±0.1 | 2.2±0.1 |
| MM | 1.0±0.1 | 1.0±0.1 | 1.0±0.1 |

**^a.^** The growth rate on CM supplemented with H_2_O_2_ was measured at 5 days. All other growth rates were measured with 10-day-old cultures grown on at room temperature. Mean and standard deviation were calculated with results from three replicates.
