# Supplementary material for: MoSfl1 Is Important for Virulence and Heat Tolerance in Magnaporthe oryzae
Source: PLoS One. 2011 May 19;6(5):e19951. doi: 10.1371/journal.pone.0019951 (PMC3098271; doi:10.1371/journal.pone.0019951)
Supplement: Table S2 — PCR primers used in this study (DOC) [file pone.0019951.s004.doc]

**Table S2. PCR primers used in this study**

| **Name** | **Sequence (5΄→3΄)** |
| --- | --- |
| SFG/1F | ctatagggcgaattgggtactcaaattggttagcaactccacgctgtcgggcaatc |
| SFG/3R | ctttataatcaccgtcatggtctttgtagtcttgcagtcgcttgcgcttcctgtca |
| NG/R | caccaccccggtgaacagctcctcgcccttgctcacttgcagtcgcttgcgcttcctgtca |
| ScF | gaccaaccccgtcatgtctttc |
| ScR | ccgagttccaagtccagcttgt |
| 1F | actctggctagtcgggactggtgtt |
| 2R | ttgacctccactagctccagccaagccgggctgtgagacgtacaacaaa |
| 3F | gaatagagtagatgccgaccgccgggttgcgagcctttactatttgcctctttc |
| 4R | ccgttatgtcctcacagacatccctac |
| YCF | ggggtaccatggctaccgccatccaaacagcg |
| YCR | acgagctctcactgcagtcgcttgcgctt |
| YSF | ggtgctggggaacatagaag |
| YSR | cttgaaagtggctgatcgtg |
| DKF | cgagagatcaagcgccgtgcctca |
| DKR | tgaggcacggcgcttgatctctcggaagttgttgtttccatgcttgaattc |
| P1F | tcctcaacccggcgccccagctgaaccaatgccaattca |
| P1R | attggttcagctggggcgccgggttgaggaggggttggc |
| P2F | cgcgccagtcgcccctagacagatgccccaggacg |
| P2R | gcatctgtctaggggcgactggcgcgttttccagctgtg |
| P3F | cagtggccttcagcacctaaccgctcagtgcctac |
| P3R | cggttaggtgctgaaggccactgagtcgagg |
| tub/QF | ccctcgtctgcacttcttca |
| tub/QR | acatctgctgggtcaactcg |
| Buf/QF | tgaggaatttgaccgtgtctttac |
| Buf/QR | aatcaggcgaccaccaatct |
| SF/QF | agacaccaccacctccacct |
| SF/QR | tcagacccccgcttctacc |
| Hp60/QF | accatcaccaaggaggacac |
| Hp60/QR | tgggtcagccataacaccac |
| Hp98/QF | atctaagggtcggcttgctc |
| Hp98/QR | atctttgccacgcttcctct |
| Hp30/QF | ggacgccaagaagggtgt |
| Hp30/QR | ctgacggggaaggtaaaggt |
| Cos1/QF | cggaaaccgaaaaccgtgag |
| Cos1/QR | tgagtttcgagctggccctg |
| Con2/QF | cttgtcgggccgcaattat |
| Con2/QF | gtcgtgagcctcgtcttc |
| Con7/QF | agtggcagcagtggagatcc |
| Con7/QR | cgcggttgggcatagaggtt |
| Htf1/QF | taactggtttcaaaaccgga |
| Htf1/QR | ccatcgctaccttctccatc |
